# The serotonin reuptake inhibitor Fluoxetine inhibits SARS-CoV-2 in human lung tissue

**DOI:** 10.1038/s41598-021-85049-0

**Published:** 2021-03-15

**Authors:** Melissa Zimniak, Luisa Kirschner, Helen Hilpert, Nina Geiger, Olga Danov, Heike Oberwinkler, Maria Steinke, Katherina Sewald, Jürgen Seibel, Jochen Bodem

**Affiliations:** 1 Institut für Virologie und Immunbiologie, Julius-Maximilians-Universität Würzburg, Würzburg, Germany; 2 Fraunhofer Institute for Toxicology and Experimental Medicine ITEM, Member of Fraunhofer International Consortium for Anti-Infective Research (iCAIR), Member of the German Center for Lung Research (DZL), Biomedical Research in Endstage and Obstructive Lung Disease (BREATH), Hannover, Germany; 3 Lehrsstuhl Für Tissue Engineering und Regenerative Medizin, Würzburg, Germany; 4 Institute of Organic Chemistry, Julius-Maximilians-Universität Würzburg, Würzburg, Germany

**Keywords:** SARS-CoV-2, Viral epidemiology, Viral infection

## Abstract

To circumvent time-consuming clinical trials, testing whether existing drugs are effective inhibitors of SARS-CoV-2, has led to the discovery of Remdesivir. We decided to follow this path and screened approved medications "off-label" against SARS-CoV-2. Fluoxetine inhibited SARS-CoV-2 at a concentration of 0.8 µg/ml significantly in these screenings, and the EC50 was determined with 387 ng/ml. Furthermore, Fluoxetine reduced viral infectivity in precision-cut human lung slices showing its activity in relevant human tissue targeted in severe infections. Fluoxetine treatment resulted in a decrease in viral protein expression. Fluoxetine is a racemate consisting of both stereoisomers, while the S-form is the dominant serotonin reuptake inhibitor. We found that both isomers show similar activity on the virus, indicating that the R-form might specifically be used for SARS-CoV-2 treatment. Fluoxetine inhibited neither Rabies virus, human respiratory syncytial virus replication nor the Human Herpesvirus 8 or Herpes simplex virus type 1 gene expression, indicating that it acts virus-specific. Moreover, since it is known that Fluoxetine inhibits cytokine release, we see the role of Fluoxetine in the treatment of SARS-CoV-2 infected patients of risk groups.

## Introduction

Starting in December 2019, SARS-CoV-2 originated in central China became a pandemic thread with more than 70,000,000 cases worldwide and more than 400,000 deaths in summer 2020 and more than 1.7 million deaths in December 2020. Despite an extraordinary effort to keep the infection at bay, the number of infections and severe clinical cases is still growing. SARS-CoV-2 is a plus-stranded, enveloped RNA virus, which encompasses 30 kilobases. SARS-CoV-2 uses the spike protein to enter the cells via angiotensin-converting enzyme 2, which is abundantly expressed in alveolar tissue. It enters the cell via pH-dependent endocytosis. The incoming RNA is directly translated into viral proteins.

In the past decades, the development of antiviral therapies has been quite successful. Direct antiviral therapies have been designed to inhibit viral polymerase, entry and integrases. They led to complete suppression of HIV-1, Hepatitis C, D, and Herpesviruses, which led to replication and even elimination of Hepatitis C from infected patients. The understanding of SARS-CoV-2 biology has vastly increased in 2020, and several substances like Remdesivir^1^, Lopinavir^2^ and Chloroquine^3^ were reported to repress viral replication in vitro. However, these compounds showed little effect on virus replication^1,4^ or led to severe adverse side effects in human patients. Furthermore, some drugs, such as ribavirin and interferon^5^, had no significant impact on patient survival rates. Currently, monoclonal antibodies such as bamlanivimab and casirivimab plus imdevimab are considered to treat mild CoV-2 cases. However, a potent direct-acting antiviral drug is still not approved.

**Figure 1:**
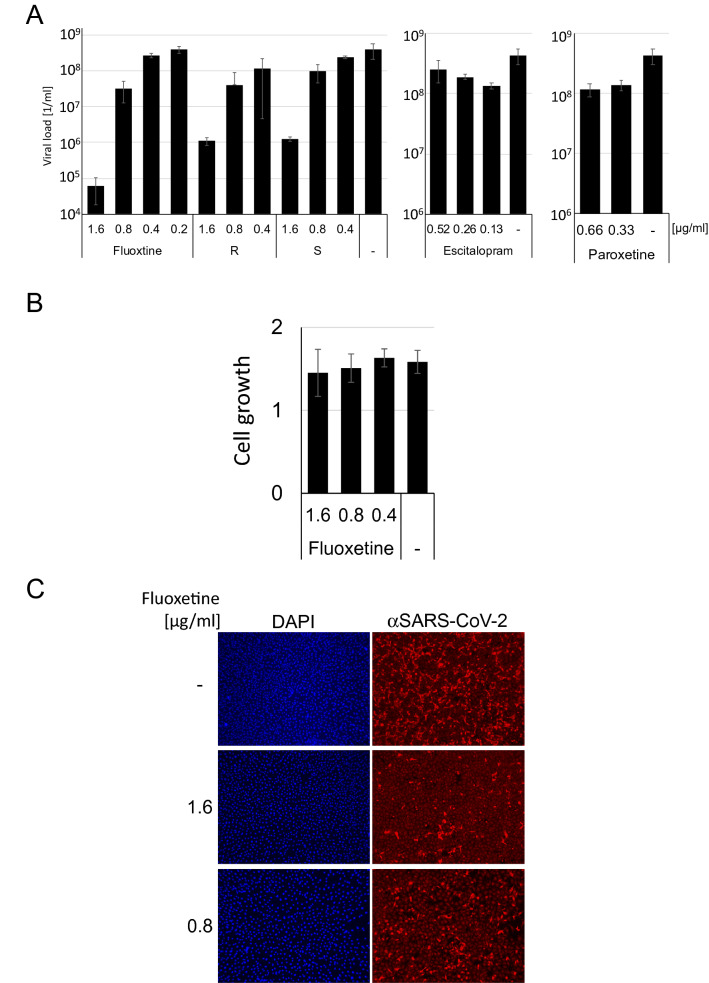
Fluoxetine inhibits SARS-CoV-2 replication. (**A**) Vero cells were incubated with the compounds and subsequently infected with SARS-CoV-2 (*S* S-stereoisomer, *R* R-stereoisomer). Cellular supernatants were collected 3 days after infection, and viral titers were determined with RTqPCR. (**B**) Fluoxetine is not toxic in Vero cells at concentrations used for the treatment of depression (0.8 µg/ml (= 2.58 µM)). Relative growth of Vero cells was determined. (**C**) Vero cells were infected with SARS-CoV-2 for 72 h, and viral proteins were detected with a SARS-CoV-2 specific antiserum (1:100) and a TexasRed-labeled donkey anti-human antibody (1:500, Dianova). The nuclei were stained with DAPI.

To circumvent time-consuming clinical trials, testing whether existing drugs are effective inhibitors of SARS-CoV-2, has led to the discovery of Remdesivir^6^. We decided to follow this path and screened approved medications "off-label"^7^ against SARS-CoV-2. In such a trial, we investigated the effect of the serotonin reuptake receptor inhibitors (SRRI) Fluoxetine, Escitalopram, and Paroxetine on viral replication. Fluoxetine blocks the serotonin reuptake transporter in the presynaptic terminal. Fluoxetine is applied at a concentration of 20 mg daily. First, cytotoxicity of the compound was analysed, and then effects on viral replication rates were measured. Vero cells were incubated with the compounds for 3 days to investigate cytotoxicity, and the relative cell growth was determined by automatic cell counting. Concentrations of 1.6 µg/ml (~ 5.17 µM) Fluoxetine did not influence cell growth. Then, Vero and Huh7 cells were incubated with the compounds at increasing concentrations and subsequently infected with patient-derived SARS-CoV-2 at an MOI of approx. 0.5. The concentrations were selected near the concentration used to treat depression (e.g., 0.8 µg/ml (~ 2.6 µM) for Fluoxetine). DMSO was used as solvent control. After 3 days, viral replication supernatants were collected, and viral RNA was extracted. Viral replication was quantified by real-time RTqPCR. All infections were performed in triplicates and repeated twice. Fluoxetine inhibited SARS-CoV-2 at a concentration of 0.8 µg/ml significantly, and the EC50 was determined with 387 ng/ml (Fig. 1A). However, it is unlikely that the serotonin reuptake transporter's direct inhibition is responsible for this suppression since neither Paroxetine nor Escitalopram interfere with viral replication at concentrations used to treat depression. Furthermore, since most of the different compounds' cellular off-target factors are similar, Fluoxetine presumably targets the viral replication directly. Fluoxetine is a racemate consisting of both stereoisomers, while the *S*-form is the dominant SSRI^8^. Thus, we analysed which stereoisomer is inhibiting SARS-CoV-2 replication. We found that both isomers show similar activity on the virus (Fig. 1A) underlining that the antiviral effect is unrelated to the serotonin reuptake receptor. To get further insights into the inhibition mechanism, we sought to visualise viral protein expression by immunofluorescence with a patient-derived antiserum. Fluoxetine treatment decreased protein expression, showing that Fluoxetine acts upstream of gene expression (Fig. 1B). To further analyse the specificity of Fluoxetine, the inhibition of other viruses was determined. Fluoxetine inhibited neither Rabies virus, human respiratory syncytial virus (RSV) replication nor the Human Herpesvirus 8 or Herpes simplex virus type 1 gene expression, indicating that it acts virus-specific (Fig. 1C).

**Figure 2:**
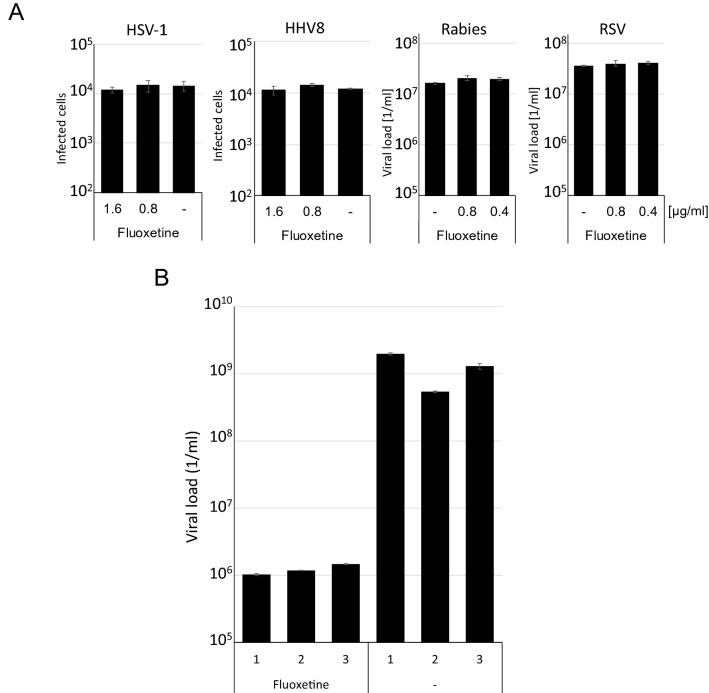
Fluoxetine inhibits virus replication specifically and SARS-CoV-2 in human lung tissue. (**A**) BHK21 and HepG2 cells were infected with either *gfp*-encoding HSV-1, HHV8, with a Rabies vaccine strain or with a patient-derived RSV. Viral titers were determined by RTqPCR (RSV, Rabies) or infected *gfp*-expressing cells were counted with an Ensight device (PerkinElmer). (**B**) Human precision-cut lung slices were treated with 1.6 µg/ml Fluoxetine infected with SARS-CoV-2. After 3 days virus, the supernatants were harvested, and viral infectivity was analysed by infecting Vero cells. The resulting viral load was determined by RTqPCR. Each bar represents the mean of 3 RTqPCR reactions from a single PCLS. The error bars represent the standard deviation.

However, other substances active in cell culture experiments did not meet antiviral CoV-2 therapy expectations in infected patients. Since neither Vero cells nor Huh7 represents a human-like tissue model, we decided to establish a patient-near infections model by infection of human precision-cut lung slices (PCLS). First, the slices were treated with Fluoxetine (1.6 µg/ml) or Lopinavir and subsequently infected with SARS-CoV-2. After 3 days, 100 µl of the culture supernatants were harvested. These supernatants were used to infect Vero cells to determine viral infectivity. We determined viral loads after 3 days of incubation by RTqPCR using 125 µl of the Vero cell culture supernatant and the dual-target SARS-CoV-2 PCR assay (Fig. 2). The PCLS allowed replication of SARS-CoV-2 since we could detect high viral infectivity from the transferred supernatants. The treatment with Fluoxetine reduced viral loads about two orders of magnitude, whereas Lopinavir failed to reduce viral titers in this assay.

Fluoxetine was introduced in clinics during the seventies and is a well-studied drug since it has been used in humans for almost four decades. Fluoxetine is used to treat depression, bulimia, panic disorders and premenstrual dysphoric disorders. Fluoxetine was shown to reduce the cytokine release, which could help control the cytokine storm, a clinical thread in severe CoV-2 cases. Clinical trials addressing this essential question will end in 2021. Furthermore, the patent of Fluoxetine has long expired. Thus, it is available from different companies, and relatively cheap. We see the role of Fluoxetine in the early treatment of SARS-CoV-2 infected patients of risk groups. This report highlights that the R-Stereoisomere of Fluoxetine represses SARS-CoV-2. It remains to be elucidated whether this stereoisomer displays less adverse side-effects.

## Methods

### Virus and antisera

The virus was obtained from the department of infectious medicine (Würzburg) and isolated for diagnostic reasons. The antisera were obtained from the blood bank (Würzburg).

### Cytotoxicity and cellular proliferation assays

The proliferation of cells was determined by direct automatic cell counting. Cells were seeded on optical plates (CellCarier-96, PerkinElmer) and counted before the experiments. Then the Fluoxetine was added, and the cells were incubated for 3 days. The cell numbers per well were determined using the PerkinElmer Ensight reader. The numbers were compared to the solvent controls.

### RNA quantification

125 µl of the cellular supernatants were harvested, and the virus was inactivated by the addition of 250 µl of the MagNA Pure LC total NA Lysis/binding buffer (Roche). Viral RNAs were isolated with the MagNa Pure 24 NA isolation device according to the manufacturer's instructions (Roche). The RNA Master Probe kit was used for amplification as described by the manufacturer (Roche). PCR-Setup was performed with the Liquid Handling Station (BRAND, Germany) in triplicate assays and quantified with the LightMix Assay SARS-CoV-2 RdRP RTqPCR assay kit (TIB MOLBIOL, Germany) and the RNA Process Control kit (Roche). The PCR was pipetted with a pipette robot device to ensure quality (BRAND, Germany). All PCRs were performed using the LightCycler 480II (Roche). Quantifications were performed with the respective cycler software.

### PCLS preparation

Only tissue from macroscopically and microscopically disease-free parts of the lung were used for experiments. Human precision-cut lung slices (PCLS) with peripheral airways were prepared as described before^9^. Briefly, a lung lobe was inflated with 2% agarose/medium solution. After the polymerisation, tissue cores of 8 mm in diameter were sliced into 300 μm thin slices. Only tissue slices containing airways with intact, full smooth-muscle layers, visible regular cilia beating, and comparable airway size as assessed by light microscopy were used in this study. Tissue slices were cultivated submerged in medium (DMEM/F12 (Life Technologies, Darmstadt, Germany) supplemented with 1% Penicillin/Streptomycin (Lonza, Verviers, Belgium)) at 37 °C, 5% CO_2_ overnight. The infection experiments were performed from a single donor and repeated with samples from a second donor.

### Ethics statement

Human lung lobes were acquired from patients undergoing lobe resection for cancer at Hannover Medical School (MHH, Hannover, Germany). The use of the tissue for research was approved by the ethics committee of the Hannover Medical School (MHH, Hannover, Germany) and is in compliance with *The Code of Ethics of the World Medical Association* (number 2701–2015). All experiments were performed in accordance with relevant guidelines and regulations. All patients gave written informed consent for the use of explanted lung tissue for research and to publish the results. No sample tissues were procured from prisoners.
